# Dual-energy index variation when evaluating the potential ferromagnetism of ex vivo bullets

**DOI:** 10.4102/sajr.v27i1.2701

**Published:** 2023-07-27

**Authors:** Francois A. van der Merwe, Eugene Loggenberg

**Affiliations:** 1 Department of Clinical Imaging Sciences, Faculty of Health Sciences, University of the Free State, Bloemfontein, South Africa

**Keywords:** dual-energy computed tomography, ferromagnetic bullets, DECT machines, inter- and intra-reader agreement, MRI safety

## Abstract

**Background:**

An MRI is potentially hazardous for patients with retained ferromagnetic bullets. Recent studies have aimed to develop dual-energy computed tomography (DECT) as a screening tool for recognising highly ferromagnetic bullets. Inconsistent findings have been ascribed to inherent CT technology differences. Previous research demonstrated significant Hounsfield unit (HU) measurement variation among single-source CT machines.

**Objectives:**

This study investigated the theoretical dual-energy index (DEI) variation between DECT machines when evaluating the potential ferromagnetic properties within the same sample of ex vivo bullets and metal phantoms.

**Method:**

An experimental ex vivo study was conducted on eight metal phantoms and 10 unused bullets individually positioned in the same Perspex head phantom and scanned on two DECT machines. Two senior radiology registrars independently recorded the HU readings, and DEI values were calculated. Statistical analysis was performed using non-parametric methods for paired data, namely the Signed Rank Test. The DEI values based on mean HU readings between the DECT machines were compared.

**Results:**

Inter- and intra-reader agreement was not statistically significant. The metal phantoms had poor interscanner agreement, with an overlap of the ferromagnetic and non-ferromagnetic ranges. The bullets had good interscanner agreement, with a similar ferromagnetic to non-ferromagnetic relationship.

**Conclusion:**

The use of DEI values negates the previous assumption that significant interscanner variability exists among different DECT technologies while assessing highly attenuative ex vivo bullets.

**Contribution:**

This investigation demonstrated that even though HU readings may be variable, the implementation of the DEI equation translates this into comparable values with good interscanner agreement.

## Introduction

South Africa has a very high violent crime burden, related to high morbidity and mortality rates. According to the South African crime statistics released for 2019–2020, firearm violence resulted in 7351 murders (of a total 21 325) and 12 718 attempted murder cases (of a total 18 635). Firearms were further implicated as the main tool of intimidation in most other crime categories and established relations with multiple murders.^1^

Law enforcement and criminal interactions are not the only source of gunshot injuries, as these may also occur during sport and hunting. Non-fatal gunshot injuries are widespread, and as a result, penetrating trauma makes up a large portion of the trauma population requiring medical care.^2,3^ Recent research highlighted gunshot wounds as a leading cause of spinal cord injuries, mostly involving younger males. Patients experienced a higher rate of adverse medical complications and morbidity, often required surgery, and had prolonged hospital admissions.^4^

Continuous advances and improvements in emergency medical care and technology will likely decrease trauma fatalities. It can reasonably be concluded that secondary evaluation techniques and residual lesion identification will become increasingly important as patients require further and definitive care.^5^

An MRI was previously deemed an absolute contraindication for patients with retained bullets, especially if these were close to vital structures. The main concerns are the potential hazards of in vivo position shift and the heating effect of the bullet within the MRI magnetic field. A recent investigation found that ferromagnetic MRI-related incidents are underreported and that there is a definite desire among radiology staff to broaden and advance the knowledge base and culture regarding the safety aspects of ferromagnetic materials related to MRI investigations.^6^ A recent study found that only highly ferromagnetic bullets can potentially shift in vivo position within MRI magnetic fields and that most bullets are non-ferromagnetic.^5^ It was concluded that bullet migration within the static magnetic field is minimal and related to the shape and orientation of the bullet.^7^ It was further shown that the potential ferromagnetic heating effect does not appear significant.^8^ This indicates that MRI might not be an absolute contraindication if the bullet (and composition) is known.^5^ Highly ferromagnetic bullets cause large amounts of MRI susceptibility artefacts, which degrade image quality and thus reduce clinical relevance.^5^

The Shellock MRI safety list is an invaluable resource if the bullet in question is known. Unfortunately, the current list of bullets is very limited and mostly rated for 1.5 Tesla (T) MRI fields. Clinical 3T and 7T MRI machines are becoming more widely utilised and the increased magnetic fields may elicit different or more dramatic interactions.^9^

Dual-energy computed tomography (DECT) has in recent years demonstrated its ability to characterise urinary calculi,^10^ quantify coronary calcification,^11^ contrast agent uptake measurements,^12^ ventilation-perfusion assessment with Xenon gas,^13^ and discriminate intracranial haemorrhage from contrast medium.^14^

Materials composed of different elemental compositions may produce very similar Hounsfield units (HUs) at a given energy, which significantly complicates the differentiation and classification of such materials. Each voxel’s HU represents a linear attenuation coefficient, which is not unique to any given material but rather a function of the material’s composition, its photon energy interaction and its mass density.^15^ There are no minimum or maximum limitations to the Hounsfield scale.^16^

The DECT can be defined as the use of attenuation measurements acquired with different energy spectra, in addition to the known changes in attenuation between those spectra, to differentiate and quantify material composition.^15^ This was initially explored and described by Godfrey Hounsfield, who stated in 1973, ‘Two pictures are taken of the same slice, one at 100 keV and the other at 140 keV … so that areas of high atomic numbers can be enhanced’.^17^ Alvarez and Macovski further investigated DECT in 1976^17^ and demonstrated that even with polyenergetic X-ray spectra, one could still separate the measured attenuation coefficients into their contributions from the principal forms of photon interaction with matter, namely photoelectric effect and Compton scatter.^18^

Forensic medicine studies have found DECT capable of differentiating between commonly found foreign materials,^19^ and identified a potential relationship between a bullet’s ferromagnetic properties and dual-energy index (DEI) value. The DECT has been proven superior to single-source CT while attempting to differentiate between high-density materials because of acquiring data sets at different X-ray beam energies.^20^

Bullets are made of a variety of materials. Traditionally, bullet cores contain lead, or an antimony-lead alloy, while bullet jackets are made of copper or gilding metal (copper-zinc alloy). Various materials are commonly used in modern bullets, including bismuth, aluminium, bronze, copper, steel, tin, tungsten, plastics and rubber.

The different high-density metals that constitute bullets might be discernible with industrial CT equipment (> 320 kV), but the radiation doses are not compatible with clinical applications in healthcare.^21^ Bullet characterisation with clinical CT machines has multiple obstacles pertaining to both artefact generation (e.g., high anatomic number, projectile size, metal interfaces, beam hardening and scatter) and reconstruction algorithms.^22^ The cupping artefact encountered with metals is a subcategory of beam hardening and scatter, leading to misinterpreting the obtained image as a representation of the bullet jacket and core. The ‘core’ portion of the bullet image is a combination of high attenuation and photon starvation, whereas the ‘jacket’ portion is the outer layer composed of scatter artefacts.^23^

Recent studies have aimed to combine DECT with the extended HU scale (EHUS) to discriminate between highly ferromagnetic and non-ferromagnetic bullets.^24,25^ The end goal is to determine MRI safety in patients with retained bullets, especially when close to vital structures. Most of these studies have identified a potential relationship, although the results have been inconsistent.

A commonly identified limiting factor has been CT technology differences. The CT machine manufacturer has been constant, but models and machine service ages differed.^20,26,27^ These studies demonstrated that highly ferromagnetic and non-ferromagnetic bullet DEI numbers clustered together within respected, although inconsistent, ranges.^20,26,27^ Interscanner variability has not been evaluated among different DECT machines utilising DEI values.

The purpose of this study was to investigate the theoretical DEI variation between different DECT machines while evaluating the potential ferromagnetic properties within the same sample of ex vivo bullets and metal phantoms.

## Materials and methods

### Study design, settings and projectiles

This experimental ex vivo study comparatively examined metal phantoms and bullets on two different DECT machines. Eight solid rod metal phantoms (12 mm diameter, 5 cm length) were investigated, representing the wide range of metals used in bullet production. The solid rods were bright bar steel, dark bar steel, tungsten, stainless steel, brass, lead, aluminium and copper. The copper rod was excluded from the study after the MRI testing, as it demonstrated strong magnetism and thus represented an alloy rather than pure copper.

Ten unused bullets of various calibres were examined (Table 1). The main elemental composition of projectiles was derived from physical examination. Specific manufacturers and composition were not available at the time of the investigation. These bullets represented commonly available firearm calibres. The individual bullet composition did not factor into the end study results, as each bullet’s magnetic properties were compared with its DEI value.

**TABLE 1:** Details of the bullets investigated.

No.	Bullet calibre	Bullet diameter (mm)	Bullet length (mm)	Bullet weight (actual) (mg)	Bullet grain (gr)	Bullet type	Weapon type	Metallic components		MDA (degrees)
								Core	Jacket	
1	0.303 British	7.9	33	9.0	140	FMJ	Rifle	Lead	Copper	0
2	0.308 Winchester	7.8	30	11.56	180	SJ	Rifle	Lead	Copper	0
3	0.243 Winchester	6.2	26.5	6.49	100	SJ	Rifle	Lead	Copper	0
4	0.224 Winchester	5.7	18	3.9	60	FMJ	Rifle	Lead	Copper	0
5	9 mm Browning	9.0	15	8.05	124	FMJ	Pistol	Lead	Copper	0
6	0.375 H&H Magnum	9.5	34.7	18.39	280	SHP	Rifle	Brass	None	15
7	0.303 British	7.9	22	8.0	123	SJ	Rifle	Steel	Copper	90
8	7 mm Remington	7.0	22	8.12	125	FMJ	Rifle	Steel	Copper	90
9	9 mm Lugar	9.0	15	7.58	117	FMJ	Pistol	Lead	Nickel	120
10	0.224 Remington	5.7	19	3.8	58	FMJ	Rifle	Steel	Copper	105

FMJ, full metal jacket; MDA, magnetic deflection angle; SJ, semi jacketed; SHP, solid hollow point.

According to suggestions from previous research, some ballistic projectile categories were excluded from the study because of size, composition, and CT spatial resolution restrictions.^28^ These were air rifle projectiles, shotgun pellets, paintballs and BB gun pellets.

Empirical ferromagnetic testing for each bullet and metal phantom was performed according to methods described in multiple previous studies concerning the deflection angle.^29^ The projectiles and metal phantoms were individually suspended on a string in front of a marked non-ferrous vertical board at the portal of an MRI machine (Philips 3T Ingenia, Universitas Academic Hospital’s Clinical Imaging Department), and the deflection angles from vertical were documented. Torque value investigation was not performed, as previous studies have indicated a linear relation between deflection angle and torque propensity.^24^

Three metal phantoms were non-ferrous, three demonstrated marked ferromagnetic properties, and one had intermediate ferromagnetic properties. The copper metal phantom was found to be a ferromagnetic copper alloy and was excluded from further testing.

Five bullets were non-ferromagnetic, one demonstrated mild ferromagnetic properties, and four were markedly ferromagnetic. Two ferromagnetic bullets had a larger deflection angle (exceeding 90°) compared with the ferromagnetic metal phantoms.

A realistic and reproducible study environment was achieved using a Perspex head phantom (large cylinder 16 cm diameter) while evaluating all metal phantoms and bullets. A central cylindrical cavity within the Perspex phantom enabled a reproducible perpendicular central placement of metal phantoms and bullets within the CT machine gantries.

### Methods and measurement

The metal phantoms and bullets were individually scanned on two different single-source DECT machines, which varied in manufacturer, production date, operational time, software and post-processing capabilities:

- General Electric CT Discovery 750 HD 64-slice installed at Universitas Academic Hospital, Clinical Imaging Sciences Department (December 2011). Acquisition and processing were performed on the accompanying workstation (AW Volume share 4). Data collection was carried out on AGFA PACS because of technical limitations on the AW Volume share workstation.
- Siemens Healthcare Somatom Definition AS+ 128-slice installed at Pelonomi Academic Hospital, Clinical Imaging Sciences Department (December 2020). Acquisition and processing were performed on the AW. Data collection was performed on the Syngo.via workstation.

The Siemens Somatom CT scanner allowed the EHUS (−10 240 to +30 710) activation during reconstruction. The GE Discovery required a complete system restart after activating the EHUS function, following which the EHUS was continuously active till manually deactivated.

Temporal sequential scanning of the entire scan volume was performed with tube strength at 100 kV and 140 kV on each metal phantom and projectile to allow for similar machine settings. This approach was used because the dual-energy programme settings did not allow for individual tube setting alterations, nor did the Siemens Somatom allow activation of the EHUS within the dual-energy programmes. Metal phantoms and bullets were scanned with the following parameters^26^: tube voltage 100 kV and 140 kV, exposure 400 mAs, rotation time 0.5 s, pitch 0.6, slice collimation 2 mm × 64 mm × 0.6 mm, slice acquisition 2 mm × 64 mm × 0.6 mm. Reconstruction was on a sharp tissue kernel with EHUS activated. Slice thickness was 1.5 mm on the Siemens Somatom and 1.25 mm on the GE Discovery. Both were set to 1 mm increments.^26^

**FIGURE 1:**
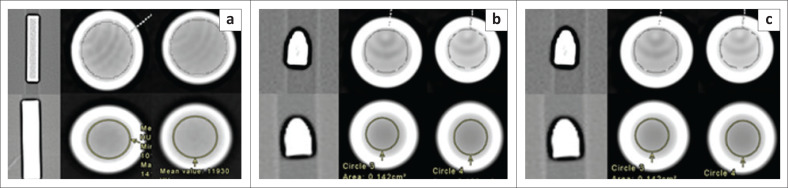
The comparative region of interest (ROI) placement for a (a) bright bar metal phantom, (b) 9 mm Browning (non-ferrous) bullet, and (c) 9 mm Lugar (ferromagnetic). Tube voltage at 100 kV (left) and 140 kV (right); Siemans Somatom (top) and GE Discovery (bottom).

The region of interest (ROI) placement^30^ was central, without incorporating the edge, and covering more than one pixel. Two sets of readings were obtained per bullet and examination, at least five slices apart. The HU readings were recorded for mean, maximum, minimum and standard deviation (s.d.). Figure 1 illustrates the comparative ROI placement for a metal phantom and 9 mm bullets.

Two independent senior radiology registrars performed all the readings, one of whom was blinded to the ferromagnetic testing. Metal phantoms and bullets were allocated a specific description used throughout the investigation period. To evaluate intra-reader agreement, a second round of scans and readings were performed on the bullets 2–4 weeks after the initial scans.

### Dual-energy index

The DECT data were reconstructed and measured using an EHUS at two different energy levels. These readings were then converted to a DEI value using a standard equation^26^:


$$DEI=\frac{Loq\;kV-High\;kV}{Low\;kV+High\;kV+2\;000}$$ [Eqn 1]

For the low kV, the EHUS reading was at 100 kV and the high kV at 140 kV.

The DEI was calculated for all measured HU readings (mean, maximum, minimum). The mean HU readings and the corresponding DEI values were submitted for statistical analyses and used for further interpretation.

### Statistical analysis

Descriptive statistics, namely frequencies and percentages for categorical data and medians and percentiles for numerical data, per type of bullet and metal phantom, were calculated. As a result of the small sample sizes, the bootstrap methodology was not appropriate; therefore, the bullet types were compared by means of non-parametric methods. Inter- and intra-rater reliabilities were calculated and described by means of non-parametric methods for paired data, namely the Signed Rank Test. Intraclass correlations (ICC) using the Shrout-Fleiss formula were calculated. The analysis was performed by the Department of Biostatistics, Faculty of the Health Sciences, University of the Free State.

### Ethical considerations

Ethical approval to conduct the study was obtained from the University of the Free State Health Sciences Research Ethics Committee (HSREC) and permission to conduct the study at a state hospital was obtained from the Free State Department of Health (ethics approval no.: UFS-HSD2020/0996/2508-0002). No consent was required in this study.

## Results

### Intra-reader and inter-reader agreement

Inter-reader agreement for the mean DEI values was not statistically significant. The Siemens Somatom had excellent ICC (*p*-values: ICC for ROI1 = 0.65:0.90900, ROI2 = 1.00:0.92990, ROI3 = 0.64:0.86502, ROI4 = 0.19:−0.00536) and the GE Discovery had good *p*-values with poor ICC (*p*-values: ICC for ROI1 = 0.43:−0.65466, ROI2 = 0.92:0.99177, ROI3 = 0.49:−0.29474, ROI4 = 0.56:−0.69093).^31^ The poor ICCs were negative and correlated with a wider s.d. on the GE Discovery. The metal phantoms demonstrated a wide *p*-value range and excellent ICC on the Siemens Somatom (*p*-values: ICC for ROI1 = 0.94:0.99720, ROI2 = 0.08:0.99856), but poor ICC for the GE Discovery (*p*-values: ICC for ROI1 = 0.47:0.38143, ROI2 = 0.02:0.78236),^31^ which had a statistically significant variation on the second ROI readings obtained on the GE Discovery.

Intra-reader agreement was not statistically significant for the bullets’ mean DEI values between the two radiology registrars (R1 and R2) with similar *p*-value ranges on the Siemens Somatom (R1 ROI1 = 0.71, ROI2 = 0.49; R2 ROI1 = 0.56, ROI2 = 0.32), and GE Discovery (R1 ROI1 = 0.06, ROI2 = 0.63; R2 ROI1 = 0.63, ROI2 = 0.2). The proposed reason for the smaller *p*-values on the GE Discovery is likely because of the wider s.d. range obtained for the mean ROIs. The metal phantoms demonstrated reduced intra-reader agreement with Investigator 2 (R2) obtaining higher *p*-values on both Siemens Somatom (R1, *p* = 0.03; R2, *p* = 0.92) and GE Discovery (R1, *p* = 0.22; R2, *p* = 0.69). Investigator 1 (R1) obtained statistically significant ROI readings on the Siemens Somatom.

### Metal phantoms

Metal phantoms (Table 2) were used to evaluate the variable DEI values pertaining to the anatomic number of different metals and demonstrated the identified cupping artefact that these high-density materials create. Metal phantoms consisted of single metal solid rods; thus, no core and jacket combination was often encountered with bullets. The obtained images dramatically demonstrated cupping artefacts, and the outer rim varied because of tube voltage and anatomic number. Cupping artefacts can serve as a visual demonstration of beam hardening and photon starvation.

**TABLE 2:** Metal phantom average Hounsfield unit and dual-energy index values.

Sample description	MDA (degrees)	Pelonomi Academic Hospital†										Universitas Academic Hospital†									
		Investigator 1 (R1)					Investigator 2 (R2)					Investigator 1 (R1)					Investigator 2 (R2)				
		100 kV	s.d.	140 kV	s.d.	DEI	100 kV	s.d.	140 kV	s.d.	DEI	100 kV	s.d.	140 kV	s.d.	DEI	100 kV	s.d.	140 kV	s.d.	DEI
Bright bar steel	90	12 795	824	12 346	512	0.0166	12 922	961	12 414	587	0.0186	11 740	1053	12 066	704	−0.0127	11 580	895	12 007	667	−0.0167
Black bar steel	90	12 675	835	12 099	461	0.0213	12 735	899	12 202	548	0.0198	12 034	1060	12 034	652	0.0000	12 025	1067	12 077	703	−0.0020
Tungsten	90	12 422	885	13 792	758	−0.0486	12 464	892	13 839	789	−0.0486	9571	1294	12 195	972	−0.1014	13 106	1212	13 390	930	−0.0264
Stainless steel	45	12 700	809	12 224	508	0.0177	12 878	969	12 252	548	0.0231	12 534	1361	12 471	1313	0.0024	12 147	1104	12 467	1310	−0.0119
Brass	0	12 246	837	13 806	724	−0.0557	12 477	994	14 053	909	−0.0552	11 259	1040	13 082	1084	−0.0692	11 407	1113	13 134	1065	−0.0651
Lead	0	12 151	773	13 669	767	−0.0546	12 428	875	13 971	867	−0.0544	11 240	828	11 992	1158	−0.0288	11 599	1093	12 793	1103	−0.0453
Aluminium	0	2350	52	2004	34	0.0545	2354	55	2008	36	0.0544	2469	25	2467	24	0.0002	2475	21	2480	20	−0.0007

DEI, dual-energy index; MDA, magnetic deflection angle; ROI, region of interest; s.d., standard deviation.

† , Average mean ROI readings.

**FIGURE 2:**
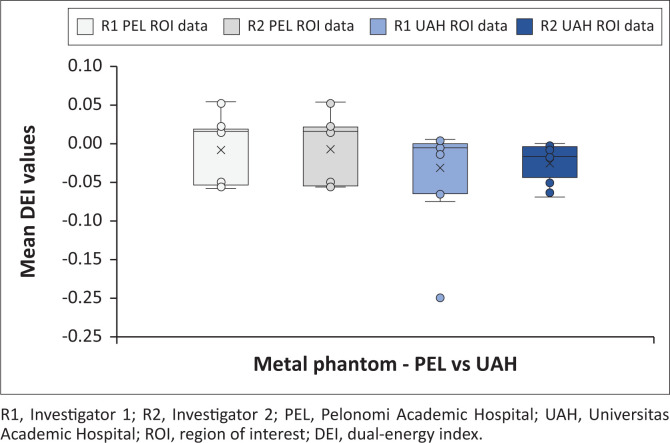
Overall findings for all metal phantom mean dual-energy index values (distinction is made between investigators and each CT machine).

**FIGURE 3:**
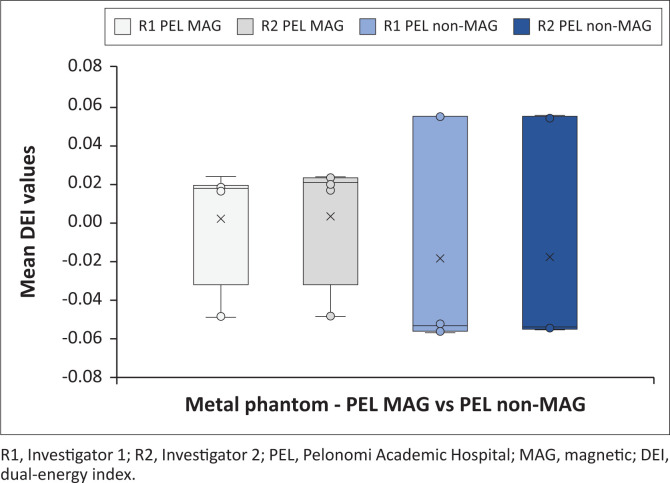
Metal phantom mean dual-energy index values obtained at Pelonomi Academic Hospital on Siemens Somatom CT machine (distinction is made between investigators and ferromagnetic properties).

**FIGURE 4:**
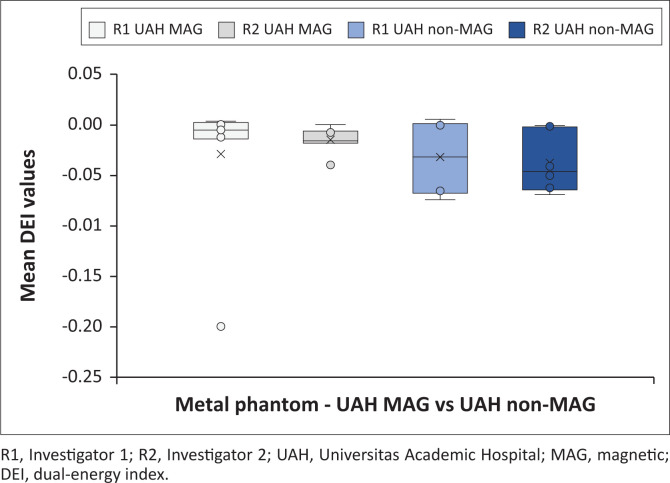
Metal phantom mean dual-energy index values obtained at Universitas Academic Hospital on GE Discovery CT machine (distinction is made between investigators and ferromagnetic properties).

Figure 2 indicates all the metal phantom mean DEI values obtained at the test sites, differentiating between investigators. Figure 3 and Figure 4 demonstrate the magnetic and non-magnetic metal phantom mean DEI values at each site, differentiating between investigators.

#### Siemens Somatom metal phantom findings

Metal phantom DEI values for both investigators were within similar ranges (R1: −0.0569 to 0.0546 with average −0.0070; R2: −0.0558 to 0.0547 with average −0.0060). Further analysis of the DEI values demonstrated that the ferromagnetic metals’ DEI ranges were centrally located within the non-ferromagnetic ranges, which is different from previous studies. The average DEI values for the ferromagnetic and non-ferromagnetic metals differed slightly (R1 ferromagnetic: −0.0488, non-ferromagnetic: −0.0569; R2 ferromagnetic: −0.0487, non-ferromagnetic: −0.0558).

#### GE Discovery metal phantom findings

Metal phantom DEI values for both investigators were once more within similar ranges, although R2 obtained a narrower range (R1: −0.0737 to 0.0064 with average −0.0299; R2: −0.0679 to 0.0006 with average −0.0240). Further analysis of the DEI values demonstrated that the ferromagnetic metals’ DEI ranges were within the upper half of non-ferromagnetic ranges, which is different from previous studies. The average DEI values for the ferromagnetic and non-ferromagnetic metals differed more pronounced (R1 ferromagnetic: −0.0279, non-ferromagnetic −0.0737; R2 ferromagnetic: −0.0142, non-ferromagnetic: −0.0679). Investigator 1 (R1) had a single significant outlier point (−0.1992), which significantly decreased the average DEI value for the ferromagnetic metals.

### Ballistic projectiles

The HU measurements (Table 3) were obtained from the central region of each bullet. The ‘jacket’ readings approached HUmax, even with the EHUS. The measured areas of each bullet and phantom depended on the overall diameter and the thickness of the beam hardening artefacts. Thus, the measured areas around the bullet centre varied between most readings.

**TABLE 3:** Bullet average Hounsfield unit and dual-energy index values.

Sample description	MDA (degrees)	Pelonomi Academic Hospital†										Universitas Academic Hospital†									
		Investigator 1 (R1)					Investigator 2 (R2)					Investigator 1 (R1)					Investigator 2 (R2)				
		100 kV	s.d.	140 kV	s.d.	DEI	100 kV	s.d.	140 kV	s.d.	DEI	100 kV	s.d.	140 kV	s.d.	DEI	100 kV	s.d.	140 kV	s.d.	DEI
Study bullet #1 (0.303 British)	0	10 320	595	9190	610	0.0896	12 318	637	12 177	715	0.0476	10 408	375	7831	345	0.1333	12 641	783	12 994	550	−0.0450
Study bullet #2 (0.308 Winchester)	0	15 665	615	17 995	603	−0.0654	16 308	879	18 508	590	−0.0546	17 483	1133	20 397	798	−0.0732	17 022	1042	19 656	637	−0.0682
Study bullet #3 (0.243 Winchester)	0	18 449	313	21 089	323	−0.0646	18 712	508	21 531	391	−0.0667	21 912	866	25 395	585	−0.0738	22 109	883	25 570	545	−0.0697
Study bullet #4 (0.224 Remington)	0	19 134	344	21 235	426	−0.0496	19 199	370	21 338	475	−0.0502	23 870	907	26 494	657	−0.0502	23 059	745	25 813	661	−0.0542
Study bullet #5 (9 mm Browning)	0	13 605	794	15 196	806	−0.0517	13 635	803	15 974	788	−0.0738	14 224	964	16 012	1031	−0.0555	14 516	1131	16 207	1180	−0.0514
Study bullet #6 (0.375 H&H Magnum)	15	13 989	825	15 501	699	−0.0481	14 244	898	15 778	838	−0.0479	14 909	1207	16 445	863	−0.0461	14 831	1375	16 522	1060	−0.0507
Study bullet #7 (0.303 British)	90	15 546	700	17 706	596	−0.0614	16 074	731	18 551	722	−0.0672	18 159	1101	21 040	1080	−0.0700	17 501	1093	20 085	999	−0.0651
Study bullet #8 (7 mm Remington)	90	15 433	640	17 576	644	−0.0612	15 682	666	17 781	679	−0.0592	18 065	1138	20 637	1048	−0.0628	16 995	958	19 591	990	−0.0680
Study bullet #9 (9 mm Lugar)	120	13 748	773	15 404	788	−0.0531	13 690	766	15 687	833	−0.0635	14 608	1054	16 764	1159	−0.0645	46 842	1064	17 024	1205	−0.0649
Study bullet #10 (0.224 Remington)	105	19 566	345	21 906	788	−0.0539	18 938	337	22 048	330	−0.0735	24 248	843	27 172	474	−0.0547	24 278	881	27 266	504	−0.0558

DEI, dual-energy index; MDA, magnetic deflection angle; ROI, region of interest; s.d., standard deviation.

† , Average mean ROI readings.

**FIGURE 5:**
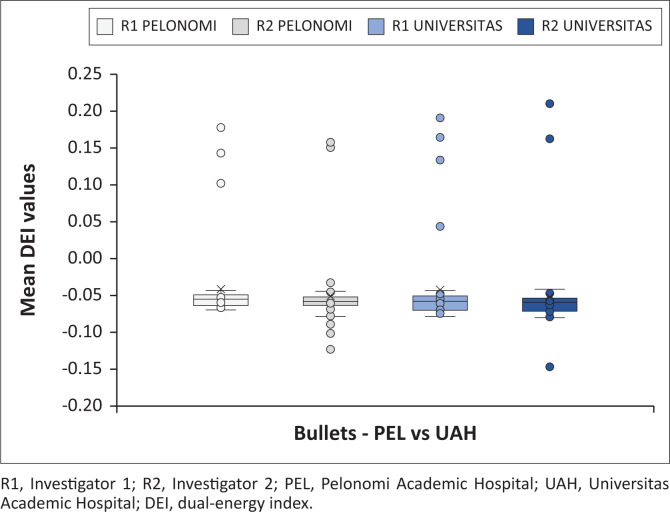
Overall findings for all bullet mean dual-energy index values (distinction is made between investigators and each CT machine).

**FIGURE 6:**
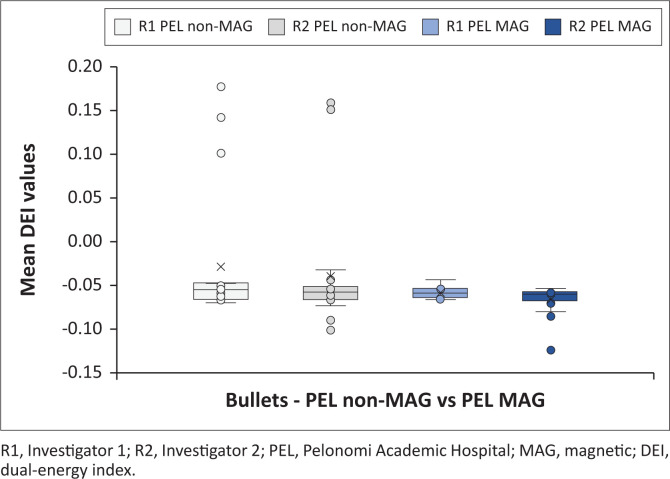
Bullet mean dual-energy index values obtained at Pelonomi Academic Hospital on Siemens Somatom CT machine (distinction is made between investigators and ferromagnetic properties).

**FIGURE 7:**
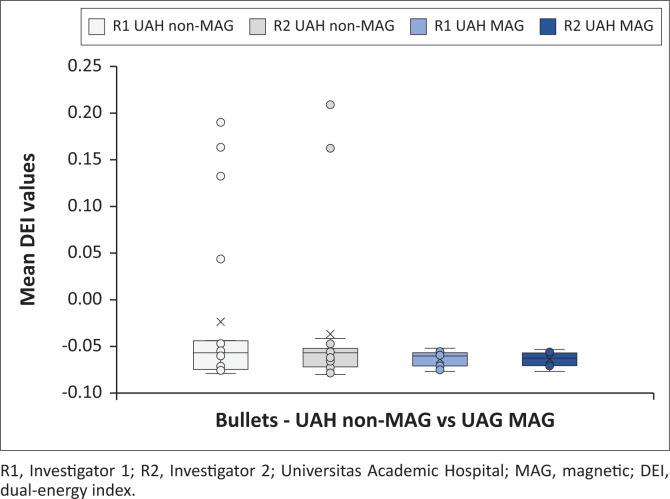
Bullet mean dual-energy index values obtained at Universitas Academic Hospital on GE Discovery CT machine. Distinction is made between investigators and ferromagnetic properties.

Figure 5 indicates all the bullet mean DEI values obtained at the test sites, differentiating between investigators. Figure 6 and Figure 7 demonstrate the magnetic and non-magnetic bullet mean DEI values at each site, differentiating between investigators.

#### Siemens Somatom bullet findings

The bullet mean DEI values of both investigators had wider ranges compared with the metal phantoms (R1: −0.0635 to 0.178 with average −0.0419; R2: −0.1239 to 0.1589 with average −0.0509). Further analysis of the DEI values demonstrated that the ferromagnetic bullet DEI ranges were within the non-ferromagnetic ranges, which was different from previous studies. The average DEI values for the ferromagnetic and non-ferromagnetic bullets differed more significantly than the metal phantoms (R1 ferromagnetic: −0.0574, non-ferromagnetic: −0.0283; R2 ferromagnetic: −0.0658, non-ferromagnetic: −0.0395). The non-ferromagnetic bullets had more pronounced positive outlier DEI values, which increased the average DEI values.

#### GE Discovery bullet findings

The bullet mean DEI values of both investigators were once more within similar ranges (R1: −0.0787 to 0.1914 with average −0.0417; R2: −0.0796 to 0.2101 with average −0.0511). Further analysis of the DEI values demonstrated that the ferromagnetic bullet DEI ranges were within the non-ferromagnetic ranges, which was different from previous studies. The average DEI values for the ferromagnetic and non-ferromagnetic bullets differed more pronounced (R1 ferromagnetic: −0.0630, non-ferromagnetic: −0.0239; R2 ferromagnetic: −0.0635, non-ferromagnetic: −0.0358). The non-ferromagnetic bullets had more pronounced positive outlier DEI values, which increased the average DEI values.

## Discussion

This study aimed to investigate the proposed interscanner variability described as a limiting factor in previous literature pertaining to DECT as a screening tool for ferromagnetic ballistic projectiles because of previous inconsistent findings.^20,26,27,30^

Ruder et al.^30^ demonstrated a considerable HU variability between different CT machines, except for brass, which was found to completely attenuate X-ray beams and thought to obscure the differences observed with other materials. Ruder et al.^30^ also showed that DECT-capable machines had the lowest HU readings among the researched CT machines, although dual-energy was not utilised. The researchers used 80 kV tube voltage and a fixed tube current time product of 130 mAs. These settings were a practical decision because of the CT machine limitations, as 80 kV was the only tube voltage available on all the machines.^30^ Paulis et al.^23^ concluded that higher tube voltages and current time products reduce artefact influences on material differentiation, following which they recommended 150 kV tube voltage and 400 mAs exposure.

The conclusion of Ruder et al.^30^ holds for single-source HU readings, and the current investigation also had variable individual HU readings among the studied DECT machines. However, the use of DEI values was found to be more consistent between the investigated DECT readings. The DEI values are functions of the HU readings and require different energy levels to calculate, which has the result that the CT technology differences are negated. It can be deduced that the CT technology differences affecting the low kV readings, similarly, influence the high kV readings and that this is annulled during the DEI value calculation.

Winklhofer et al.^20^ concluded that DECT was more reliable at differentiating materials than single-energy CT. They found that the optimal tube voltage combination was 100 kV/140 kV, although exposure was adjusted to produce constant radiation doses. Diallo et al.^26^ used the same scan parameters and CT manufacturers, although the models differed (Somatom Definition Flash vs Somatom AS+). Both studies identified a DEI difference between ferromagnetic and non-ferromagnetic bullets, although the relations were opposite. Multiple other research studies^19,20,23,26,27,30^ have been conducted using Siemens models, which do not account for the proposed inter-manufacturer variability. The reason for the variability among results has been ascribed to individual machine differences.

Our investigation was conducted with two completely different DECT machines, which currently appears to be the only study to utilise other manufacturers. We believe the machines represent a broad enough CT technology variation to represent the proposed limitations previously identified in the tabled articles. Our research advances the literature by evaluating this previously suggested interscanner variability.

The machines had variable DEI ranges for the metal phantoms (Figure 2, Figure 3 and Figure 4), with considerable overlap. The Siemens Somatom DEI averaged more positive (−0.0065) compared with the GE Discovery (−0.027), although the latter had narrower ranges. The ferromagnetic phantom DEI ranges (Siemens Somatom average: −0.0488; GE Discovery average: −0.0211) were within the non-ferromagnetic phantoms’ DEI ranges (Siemens Somatom average: −0.0564; GE Discovery average: −0.0708). This is further complicated by the Siemens ferromagnetic readings occupying the middle ranges and the GE ferromagnetic readings occupying the upper half of the non-ferromagnetic ranges. These findings indicate that the machines have a significant variation, and the individual machines might not be able to clearly distinguish ferromagnetic from non-ferromagnetic metals without individually calibrated reference ranges.

The bullets (Figure 5, Figure 6 and Figure 7) had a much better average DEI value agreement between the two DECT machines, with even the outlier points demonstrating similar values. The bullets showed a much narrower DEI range, with a similar ferromagnetic to non-ferromagnetic relationship. The average ferromagnetic DEI values were significantly lower than the average non-ferromagnetic values, similar to Diallo et al.^26^ and the opposite to Winklhofer et al.^30^ The current findings demonstrate a more pronounced overlap of DEI values while comparing ferromagnetic to non-ferromagnetic bullets, whereas previous studies had a more pronounced difference with less overlap.

Despite these variations with previous similar research, the main objective of this investigation was to assess variability between different DECT machines. The average bullet DEI values were comparable between the machines (Siemens Somatom: −0.0464; GE Discovery: −0.0464). Furthermore, even the average ferromagnetic (Siemens Somatom: −0.0616; GE Discovery: −0.0633) and non-ferromagnetic (Siemens Somatom: −0.0339; GE Discovery: −0.0299) bullet DEI values were comparable and within close proximity to each other. The average DEI values give a false impression of a wide difference between ferromagnetic and non-ferromagnetic values, as the ranges largely overlap. Significant outlier values for the non-ferromagnetic bullets increase the average DEI values, however, the outlier DEI values are similar between the DECT scanners.

### Limitations

This research investigation had a couple of limitations worth mentioning and may require future study. Small projectiles and fragments were excluded based on CT spatial resolution limitations.^20^ Core readings were used for DEI value calculation and analysis, as the ‘jackets’ have previously been shown to be unhelpful.^20,23^ Undamaged unused bullets were used, and optimally placed within the centre of the gantries at perpendicular angles to the X-ray beams. This was done to limit projectile variables and test the research question in isolation. We are aware that bullets will orientate randomly and deform upon impact, and the position related to the gantry and surrounding structures may be different in real life situations. The metal phantom and bullet samples were small; however, we believe that these were sufficiently representative. Larger metal phantom and projectile samples may have increased statistical relevance, although larger studies have been conducted on single machines. Finally, ex vivo analysis is still under investigation and not yet applicable to emergency radiology. Further research and agreement are required before implementation on the living should be considered.

## Conclusion

In conclusion, the use of DEI values negates the previous assumption that significant interscanner variability exists among different DECT technologies while assessing highly attenuative ex vivo ballistic projectiles. Previous research indicated that different HU measurements are obtained with different CT machines, but this has not yet been reassessed on DECT machines utilising DEI values. This investigation has demonstrated that even though HU readings may be variable, the implementation of the DEI equation translates this into comparable values with good interscanner agreement.

Future research on DECT implementation for MRI safety prediction related to bullets should use DEI as a reference rather than HU only.
